# Older Couples Coping With Adverse Life Events: The Importance of Dyadic Personality Profiles

**DOI:** 10.1093/geroni/igaa057.1942

**Published:** 2020-12-16

**Authors:** Shuangshuang Wang, Kyungmin Kim, Jeffrey Burr

**Affiliations:** 1 Shandong University, Jinan, Shandong, China; 2 University of Massachusetts Boston, Boston, Massachusetts, United States

## Abstract

Personality can be an important resource as older couples cope with adverse life events. Analyzing 4,893 older couples from the Health and Retirement Study, this study examined how one’s own and spouse’s adverse life events (health decline, job exit, loss of wealth, family member’s death) occurring in the past two years are associated with changes in depressive symptoms. We further examined the moderating effects for this association of six dyadic personality profiles (combinations of spouses’ positive and negative personality characteristics). We found significant actor and partner effects of health decline for increases in both spouses’ depressive symptoms, and significant actor effects of a family death for husbands’ increased depressive symptoms. For wives, having positive personality profiles buffered negative effects of one’s own health decline and spouses’ family death, whereas having negative profiles intensified negative effects of husbands’ job exit and loss of wealth on the depressive symptoms for both spouses.

